# Effect of TRC and F/TRC Strengthening on the Cracking Behaviour of RC Beams in Bending

**DOI:** 10.3390/ma14174863

**Published:** 2021-08-27

**Authors:** Edoardo Rossi, Norbert Randl, Tamás Mészöly, Peter Harsányi

**Affiliations:** Faculty of Civil Engineering and Architecture, Carinthia University of Applied Science (CUAS), Villacher Straße 1, A-9800 Spittal an der Drau, Carinthia, Austria; n.randl@cuas.at (N.R.); t.meszoely@cuas.at (T.M.); p.harsanyi@cuas.at (P.H.)

**Keywords:** textile-reinforced concrete (TRC), fibre/textile-reinforced concrete (F/TRC), strengthening, cracking behaviour, serviceability limit state, digital image correlation (DIC)

## Abstract

The increasing demand on the performance of existing structures, together with their degradation, is among the main drivers towards the development of innovative strengthening solutions. While such solutions are generally aimed at increasing the load-bearing capacity of structural elements, serviceability limit states also play an important role in ensuring the performance and durability of the structure. An experimental campaign was performed to assess the cracking behaviour of reinforced concrete beams strengthened with different typologies of Textile-Reinforced Concrete. The specimens were monitored using Digital Image Correlation (DIC) technology in order to obtain a quantitative evaluation of the evolution of the crack pattern throughout the whole test. Results show the beneficial effects of this retrofitting strategy both at ultimate limit states and serviceability limit states, provide detailed insights on the progression of damage in the specimens and highlight how different parameters impact the cracking behaviour of the tested elements.

## 1. Introduction

Degradation of concrete constructions is an important issue that seriously affects the durability and functionality of structures. The costs related to maintenance, rehabilitation and upgrade of the existing built environment are extremely high. According to a 2013 ASCE report [1], approximately 11% of US bridges were structurally deficient and 25% functionally obsolete. According to the Federal Highway Administration (FHWA), the replacement and repair cost of the deficient bridges alone was almost USD 76 billion, while the investment backlog for the nation’s bridges amounted to USD 121 billion. Similar scenarios can be found worldwide as a consequence of structures’ aging and increase in performance demand, such as increased traffic levels. According to a 2004 study [2], 16% of European railway bridges were between 50 and 100 years old, while 55% were between 20 and 50 years old, thus resulting in elevated maintenance and upgrade costs. Germany, as an example, planned in 2018 an investment of EUR 3.9 billion, expected to increase to EUR 4.4 billion in 2021, for the maintenance of federal highways, 37% of which was exclusively for bridge maintenance [3].

A promising technique for the rehabilitation and strengthening of concrete structures consists in the use of Textile-Reinforced Concrete (TRC). Such material, also known as Textile-Reinforced Mortar (TRM) or Fabric-Reinforced Cementitious Matrix (FRCM), is composed of a high strength fabric embedded in a cementitious matrix. Several studies have been performed on the application of such materials, both on concrete and masonry elements, showing very promising results [4,5,6,7,8,9,10,11]. More recently, researchers have been studying an enhanced version of these kinds of materials, consisting of the admixture of short dispersed fibres in the cementitious matrix used to bind the textile [12,13,14,15,16,17,18,19,20,21]. Such material will be referred to in the present work as Fibre/Textile-Reinforced Concrete.

Most of the studies performed on such strengthening solutions, however, focus mainly on the increase in load-carrying capacity of the retrofitted element. Only a few works reported a detailed description of the evolution of the crack pattern of the strengthened elements. Yin et al. [22] analysed beams strengthened with TRC and subjected to four-point bending focusing on the effect of such strengthening solution in terms of cracking load and maximum crack width. They observed an increase in the cracking load ranging between 11% and 31%, depending on the number of textile layers, presence of short dispersed fibres and mechanical anchorage elements. In terms of maximum crack width at the yielding load, they recorded a reduction ranging between 57% and 78%. Verbruggen et al. [23,24] performed four-point-bending tests on reinforced concrete beams strengthened both with TRC (using randomly oriented glass fibre mats) and CFRP (carbon fibre-reinforced polymer) strips. The crack pattern and its evolution were analysed using a Digital Image Correlation (DIC) system and an Acoustic Emission (AE) system which enabled the characterization of every crack in the pure bending zone in terms of crack width and horizontal displacement of the tensile chord. They observed that the TRC elements had a beneficial effect on the cracking behaviour of the specimens, increasing the number of cracks while reducing their opening. From the comparison of the two publications, it can also be observed that the geometry of the specimens and strengthening elements influences the cracking behaviour. In a subsequent study [25], the effect of the width of the TRC strengthening element was investigated. The results confirmed such dependency upon the geometry, recording an increased number of cracks and reduced crack width for specimens strengthened with wider TRC elements. Park et al. [26] instead focused on the number of cracks that appeared on the specimens, once again tested in a four-point-bending setup. They analysed both cracks on the whole length of the specimens and the ones that appeared in the pure bending zone. They observed that specimens strengthened with TRC tended to concentrate the cracks in the pure bending zone, resulting in an advantageous distribution.

The following work presents a detailed analysis of the evolution of the crack pattern of Reinforced Concrete (RC) beams strengthened with different typologies of TRC and F/TRC, varying in terms of cementitious material and number of textile layers. Different typologies of mechanical anchors were also installed on some of the specimens. The reported results complement a previous work [20] focused on the analysis of such strengthening solutions in terms of Ultimate Limit State (ULS) and increase in load-carrying capacity. In this work, it is shown how the different parameters influence the evolution of the crack pattern. The cracks were analysed in terms of Crack Opening Displacement (COD), monitoring the evolution of both the maximum and average COD with load, and number of cracks. Furthermore, to assess the performance of the members at Serviceability Limit States (SLS), two representative load levels were chosen and the cracking state was analysed and discussed.

## 2. Materials and Methods

### 2.1. Geometry and Test Setup

**Figure 1:**
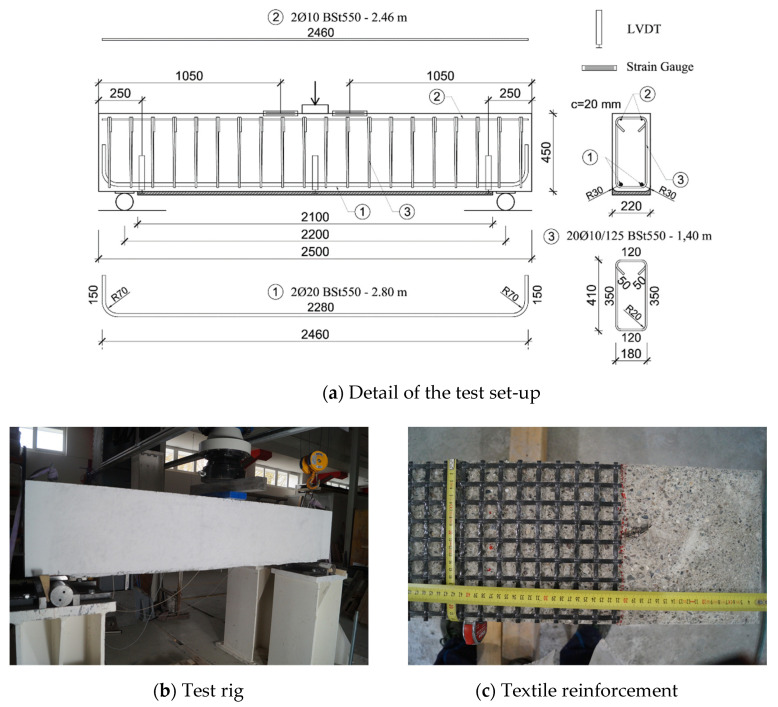
Geometry and reinforcement of the beam specimens.

A total of 11 beams were strengthened with TRC or F/TRC elements differing in the number of textile layers, typology of concrete and mechanical anchorage configuration. Two more beams were tested as a reference. Each beam was 2.5 m long and had a cross-section of 220 × 450 mm. The internal longitudinal reinforcement consisted of 2 Ø20 steel bars located in the tensile zone and 2 Ø10 steel bars in the compression zone. A total of 20 Ø10 stirrups, with a spacing of 125 mm, were used as transversal reinforcement. The strengthening elements were cast, using different methods (lamination, manual pouring and pumping), against the soffit of the beam over a length of 2.1 m. In order to ensure a good bond between the beams and the strengthening elements, the concrete surface was roughened using a waterjet gun until an average roughness of approximately 1.5 mm, measured with the sand patch method, was obtained. A detailed representation of the specimens can be found in Figure 1a.

All beams were tested using a three-point bending setup, with the supports, consisting of two steel rollers placed at a distance of 2.2 m, thus resulting in a distance of 50 mm from the strengthening layers. A detail of the test setup can be found in Figure 1a, while Figure 1b,c show, respectively, a beam under the test rig, and a detail of the textile. The load introduction system consisted of a hydraulic cylinder, mounted on a steel frame, with a maximum load capacity of 2100 kN and a resolution of 0.01 kN.

Two sets of measuring devices were used to monitor the deformative state of the specimens. The first, more traditional one, consisted of a series of strain gauges and LVDTs (Linear Variable Displacement Transducers). Two strain gauges were placed on top of the compression zone, near the load introduction point, at a distance of 1.05 m from the ends of the beam. One LVDT was placed at the middle of the span to record the deflection. Two more LVDTs were placed at each end of the strengthening layer, fixed on the substrate with the measuring tip resting on aluminium plates which were glued to the strengthening layer. They were used to monitor delamination phenomena. A schematic representation of such measuring instrumentation set can be found in Figure 1a. This set was used to obtain a live evaluation of the behaviour of the specimens during the tests and to assist in a more detailed analysis.

The second set of measuring equipment consisted of a DIC system. Such a solution was chosen due to its ability to accurately monitor the deformative state of the whole surface of the specimens, providing more insights than traditional methods. The reliability of such measuring technology was validated in previous research activities [27,28,29,30].

### 2.2. Material Characteristics and Strengthening Solutions

The textile material was the same for every specimen and consisted of one to three layers of a commercially available carbon grid characterised by a 25 mm distance between the rovings’ axes, a cross section of the strand of 3.62 mm^2^ and a textile cross-section of 142 mm^2^/m [31]. Two different cementitious materials were used to bind the textile to the specimens. One was a commercially available fine-grain pre-mixed High-Performance Concrete (HPC), while the other was a self-developed UHPFRC (Ultra High Performance Fibre Reinforced Concrete) recipe based on the results obtained in previous research [32] and also successfully used for strengthening flat slabs, however with prefabricated UHPFRC elements [33]. This particular HPC was chosen due to the possibility of applying it by means of lamination, thus avoiding the need for formwork, and because it is explicitly mentioned and mandatory to use in the German technical approval for strengthening with TRC [34]. The UHPFRC material was chosen due to its self-compacting characteristics, higher compressive and tensile strengths, and good bonding performance [35]. The fibres used in such mixture (2.5 vol%), were 5 mm long and had a diameter of 0.15 mm, resulting in an aspect ratio of 33.3. From the basis of these two materials, two additional cementitious matrices were employed: the first one, referred to as HPFRC (High-Performance Fibre-Reinforced Concrete), consists of the admixture of the short dispersed steel fibres with HPC; the second one, referred as UHPC (Ultra High Performance Concrete), has the same components of UHPFRC, without however the steel fibres. The strengthening layer had a length of 2.1 m and a width of 220 mm. Each textile layer consisted of 9 longitudinal yarns distanced 25 mm from each other’s axis. The distance between two adjacent textile layers, as well as the distance separating the layer from the concrete substrate or covering the outmost external one, was approximately 5 mm.

**Figure 2:**
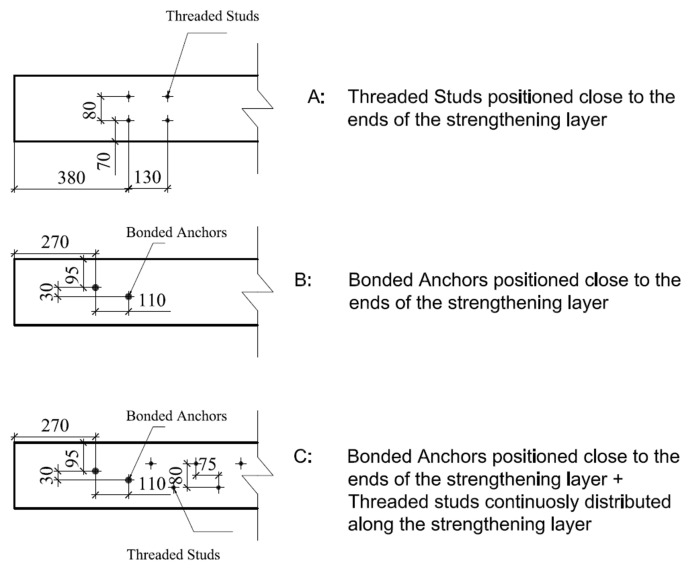
Geometrical layout of the mechanical anchorages.

Two different kinds of mechanical anchorages were also used. The first ones were short threaded studs, with a diameter of 8 mm and a total length of 62 mm, including 37 mm of embedment length. Such devices were directly shot into the substrate concrete after predrilling a hole of 23 mm depth and 5 mm in diameter. The second ones were resin bonded steel anchors with a diameter of 12 mm and a total length of 220 mm, 160 mm of which were embedded in the substrate. The mechanical anchorages were arranged in three different configurations (Figure 2). Configuration A consisted of four threaded studs positioned 380 mm away from each end of the beams. They had a longitudinal and transversal spacing of 130 mm and 80 mm, respectively. Configuration B consisted of two steel anchors, distanced 110 mm in the longitudinal direction and 30 mm in the transversal one, at each end of the beams. They were placed in a point-symmetrical manner, with the external one at a distance of 270 mm from the end of the beam. Configuration C consisted of the steel anchors positioned as in configuration B with the addition of threaded studs, arranged in a triangular pattern with a distance of approximately 110 mm, covering the remaining span.

The specimens were strengthened using two different application procedures. The first one, involving HPC and HPFRC, was performed with the beams resting on two elevated supports while alternative layers of the cementitious compound and textile fabrics were applied on the bottom side of the beam through lamination. The second one, instead, made use of a formwork holding the textile layers and in which the UHPFRC or UHPC was pumped using a modified snail pump. One specimen strengthened with UHPFRC was also produced without the use of such a pump (manually pouring the fresh concrete in the formwork) in order to assess any influence of the pumping operations. A detailed summary of the strengthening solutions tested in this experimental campaign can be found in Table 1, together with the cube compressive and splitting tensile strengths of the cementitious compounds used to produce the TRC and F/TRC elements (with the exception of the reference beams where the value refers to the substrate concrete). The specimens’ ID reflects the different strengthening solutions that were employed. It consists of up to three groups of letters/numbers. The first one (FR—Fibre-Reinforced) indicates F/TRC materials; the second one indicates both the type of cementitious matrix (P—HPC, U—UHPC) and the number of textile layers; the third indicates the configuration of the mechanical anchorage, with the only exception of specimen FR-U1-M, where the last letter refers to the application procedure (M—Manual Pouring). As an example, specimen FR-P2-B refers to a beam strengthened with F/TRC using an HP(FR)C matrix, two layers of textile fabrics and anchorages as in configuration B.

The normal strength concrete (NSC) used to produce the beams was ordered from an external company and was supposed to be a C30/37; however, a higher quality product was delivered, closer to a C40/50. The material of the steel reinforcing bars was a B550 B with a tensile yield strength close to 600 MPa. Both the compressive and tensile tests on the concrete specimens were performed the same day as the beam test. The NSC compressive properties were obtained by testing 150 × 150 × 150 mm cubes. The high compression strength of the compounds used to produce the TRC and F/TRC elements was, instead, obtained using 100 × 100 × 100 mm cubes. The splitting tensile tests were performed on 100 × 200 mm cylinders. Furthermore, in order to ensure that shrinkage of the strengthening elements would not significantly affect the behaviour of the specimens (see, e.g., [36,37]), the beams were carefully inspected and no sign of distress or shrinkage cracks could be detected.

The mechanical and geometrical properties of the textile fabrics, according to its technical sheet, are the following: 3100 MPa and 3300 MPa average tensile strength for the longitudinal and transversal rovings, respectively; and modulus of elasticity greater than 220 GPa and 205 GPa for the longitudinal and transversal rovings, respectively [31].

## 3. Results

### 3.1. Load Carrying Capacity and Failure Modes

As already mentioned, a detailed discussion on how the different parameters that characterize the tested strengthening solutions affect the load-carrying capacity of the beam specimens was presented in a dedicated article [20]. Nevertheless, such results are briefly summarized here to provide a more comprehensive description of the behaviour of the beams and enable a more thorough analysis of the evolution of the crack pattern. All the beams failed in bending, due to the failure of the strengthening layers or, in the case of the reference beams, rupture of one of the longitudinal steel reinforcing bars placed in the tensile zone. A significant degradation of the compression zone was also observed in every specimen. The strengthened specimens exhibited different failure mechanisms that can be summarised as follows:

(a) rupture of the textile fabric;
(b) pull-out of the longitudinal strands from the cementitious matrix;
(c) debonding of the TRC of F/TRC element signalled by the presence of a long horizontal crack at the interface with the NSC;
(d) interlaminar shearing, where a horizontal crack formed along the textile fabric within the strengthening layer;
(e) peeling of the concrete cover, when the debonding of the strengthening element did not happen at the interface between the two materials but inside the NSC, approximately at the height of the longitudinal reinforcing bars.

**Figure 3:**
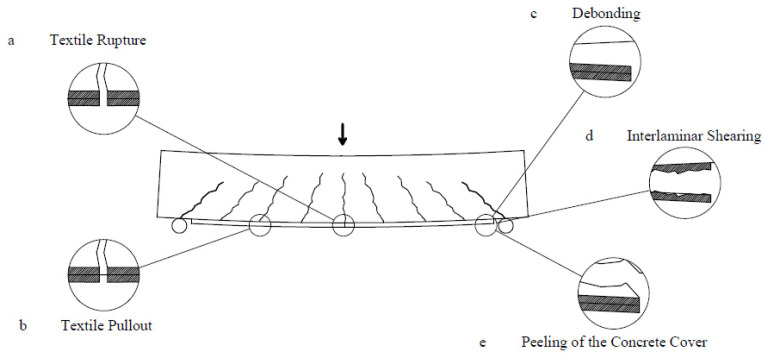
Failure modes of retrofitted beams.

A schematic representation of the observed failure modes is reported in Figure 3. The results of the test series, in terms of loads associated with the appearance of the first 0.1 mm crack (cracking load), the yielding of the steel reinforcement (yielding load) and maximum loads, together with the observed failure modes, can be found in Table 2.

All the strengthened beams showed an increase both in the yielding and in the maximum loads, ranging between 22.01 kN (P1) and 49.55 kN (P3-A) in the case of yielding loads, and between 19.14 kN (P1) and 153.37 kN (FR-U2-B) in the case of maximum loads, with a more pronounced increase for the specimens strengthened with UHPFRC. Additionally, the introduction of mechanical anchorage systems had, in most cases, a positive effect on the load-carrying capacity of the beams. Such increase is particularly noticeable when the bonded anchors were used, resulting in a gain of the load-carrying capacity between 40.7% and 48%, while, on average, the others solutions experienced an increase of 17.4%.

In terms of failure modes, it can be observed that the beams strengthened with HPC always exhibited interlaminar shearing and, in the case of the specimens without bonded anchors, such distress was the main phenomenon that led to failure. Beams strengthened with UHPC and UHPFRC, on the other hand, exhibited a more diverse pattern of failure modes. When only one textile layer, combined with pumped UHPC or UHPFRC, was used, the main failure mode was rupture of the textile. When, instead, the F/TRC element was applied by manually pouring the fresh concrete (beam FR-U1-M), the failure mode switched to debonding. All the beams with bonded anchors failed by textile pull-out, combined with interlaminar shearing and debonding of the strengthening element, showing that such anchors were able to effectively redistribute the load into the substructure even with a high degree of distress.

### 3.2. Damage Evolution and Crack Patterns

**Figure 4:**
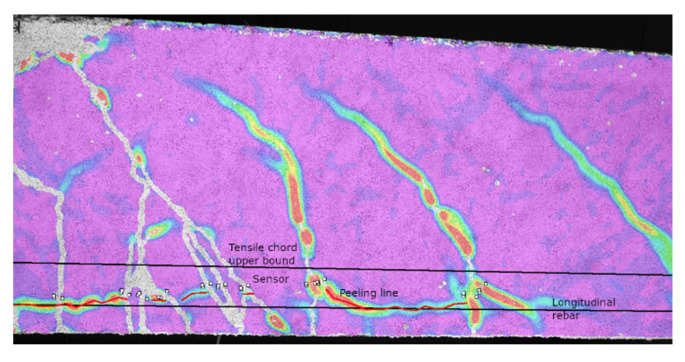
Sensor placement.

With the DIC system, it was possible to obtain detailed results on the damage evolution of the beam specimens. Such analysis was performed measuring each crack that appeared on the specimens together with their progressive opening until the maximum load was reached. The cracks were measured by means of virtual sensors just above the height where peeling of the concrete cover would take place, highlighted by a nearly horizontal strain concentration visible from the DIC measurements (Figure 4). Such measurement location was chosen since it is approximately located in the middle of the effective tension zone as defined in Eurocode 2 [38]. Furthermore, several authors suggested that corrosion phenomena might be related to the cracks crossing the reinforcement [39,40,41]. Several threshold values, representative of the damage state in the beams, were defined. These values, corresponding to crack openings of 0.1 mm, 0.2 mm, 0.3 mm and 0.4 mm, were chosen since they are the limits imposed by several international codes for the protection of concrete structural elements and depend on the aggressiveness of the environment [38,42,43].

**Figure 5:**
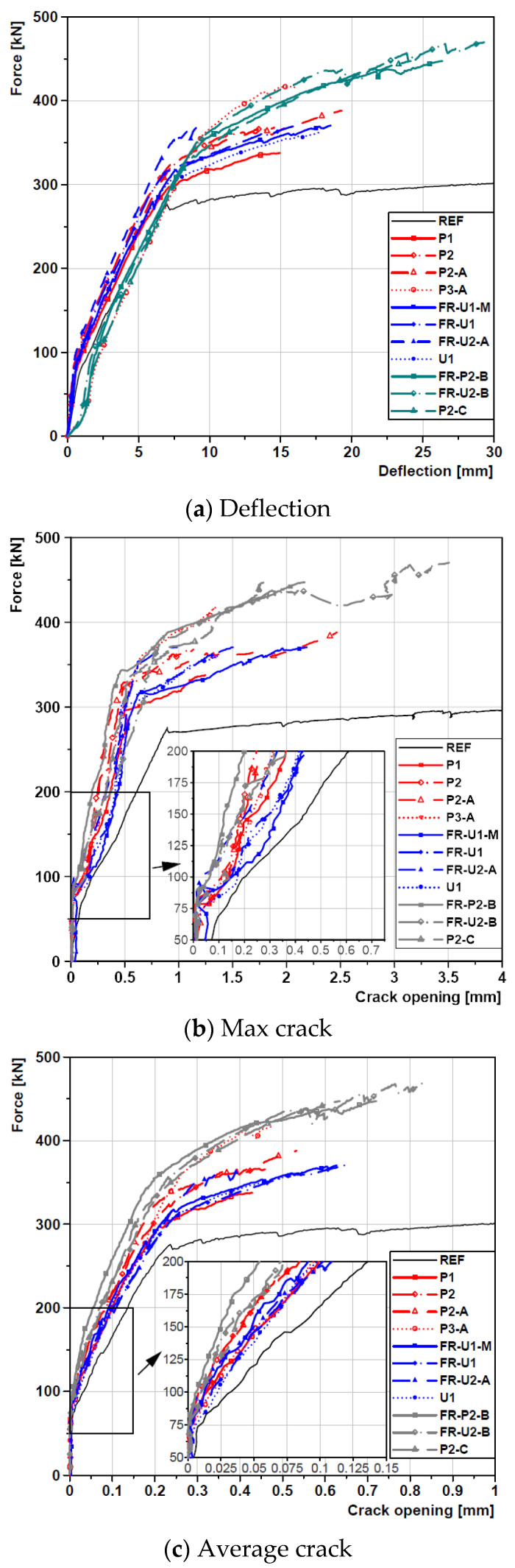
Force—Deflection/Crack Opening curves.

The crack evolution was monitored both in terms of maximum and average crack opening. Figure 5 reports such data, together with deflection, plotted against load. All the curves showing crack opening can be divided into three stages: the first stage, where the structure can be considered substantially uncracked—in this stage, several micro-cracks are formed but crack opening does not increase with load; the second stage, where cracks start to open with increasing load; and the third stage, where yielding of the steel reinforcing bars occurs and there is a strong increase in the crack opening rate. Each specimen entered the second stage approximately at 75 kN and every strengthening solution resulted in an important reduction in both crack opening at a given load and crack opening rate.

In terms of crack control capability of the strengthening solutions, all beams show the effectiveness of such type of subsequently attached F/TRC layers. In detail, however, the beams strengthened with textile-reinforced UHPC or UHPFRC show a less effective crack control capability when compared to the ones strengthened with HPC, both in terms of maximum and average crack opening, with a more pronounced difference in the former case. Particularly noticeable is the behaviour of the beams where the bonded anchors were installed, which exhibited the overall best behaviour, showing that, even at low loads, these elements enabled a good stress redistribution between the beams and the strengthening layers.

**Figure 6:**
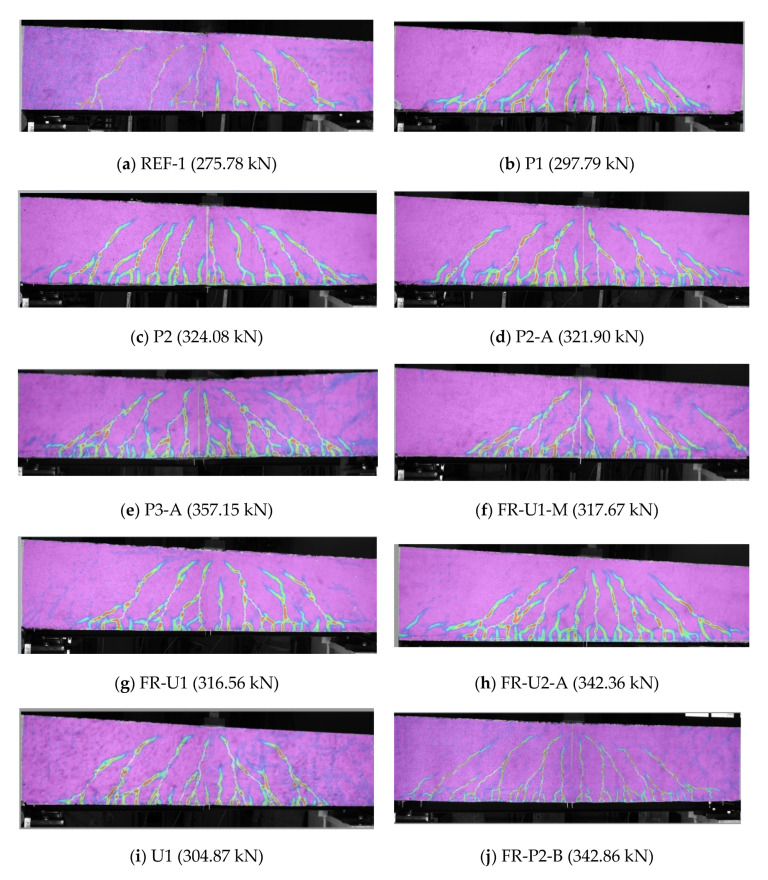

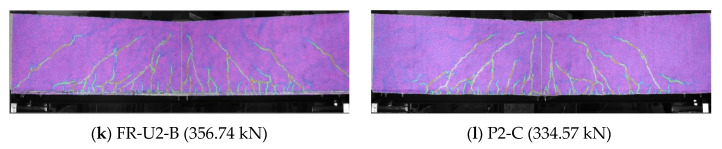
Crack patterns of all tested beams at the steel yielding load highlighted by the major principal strain.

The crack pattern of each specimen can be observed in Figure 6. Each picture was taken from the DIC evaluation at a load level corresponding to the yielding of the longitudinal steel reinforcing bars and the colours represent the major principal strain. It is important to note that the results reported in Figure 6 are only meant to show the crack pattern and, the colour scale was calibrated only to highlight the cracks.

#### Number of Cracks

**Figure 7:**
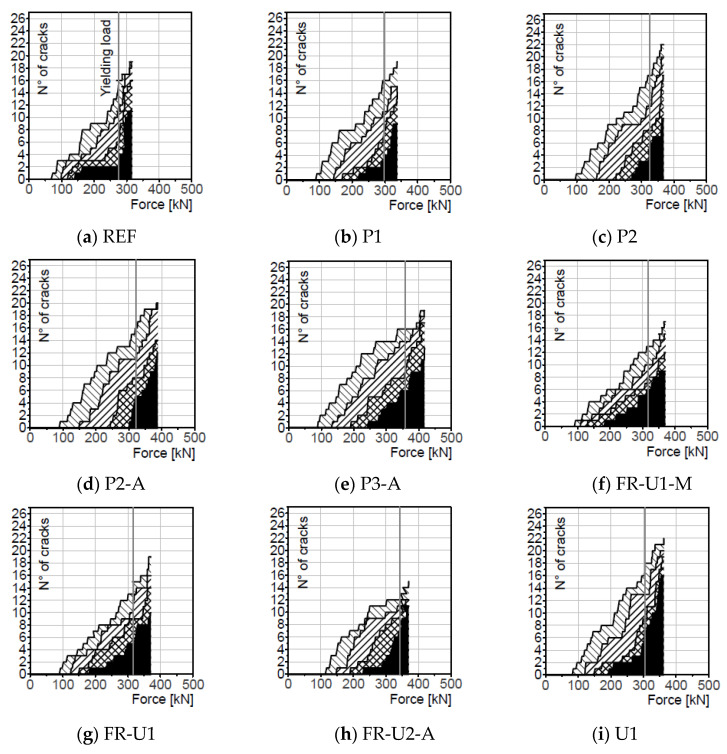

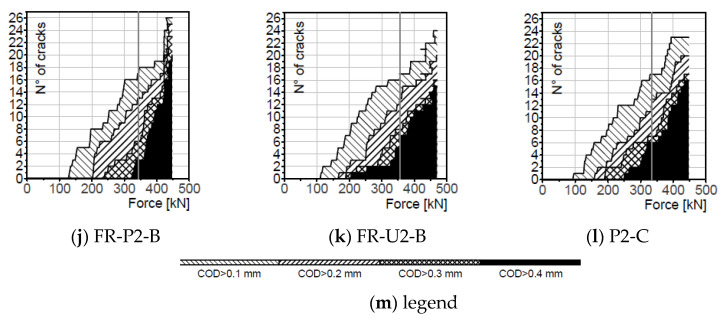
N° of cracks—Force diagrams.

The results are reported in Figure 7. A crack was counted when its opening overcame the thresholds previously described, furthermore, it is important to mention that crack closure, which was sometimes observed and probably caused by stress redistribution phenomena, was not taken into consideration. The graphs of Figure 7 report the total number of cracks that appeared on the specimens and that were larger than the reference values. The number of cracks that had an opening between two threshold values can be calculated by subtracting the respective curves.

The reference beam showed a nearly linear increase in the number of smaller cracks (0.1 mm and 0.2 mm) while the larger cracks (>0.3 mm) tended to form after yielding the steel reinforcement. Upon reaching the maximum load, nearly all the cracks reached an opening bigger than 0.3 mm and more than half overcame the threshold of 0.4 mm. Specimen P1 exhibited a more pronounced increase in the larger cracks upon reaching the yielding load and a high percentage of cracks overcoming the 0.3 mm and 0.4 mm thresholds. The formation of the larger cracks happened at significantly higher load levels. Referring to specimen P2, the smaller cracks occurred at a load level comparable to beam P1 while the larger ones formed at significantly higher loads. They started forming before the yielding load and their increase rate was nearly linear and not affected by the yielding of the reinforcing bars. Similar consideration can also be taken for beam P2-A, where 0.1 mm and 0.2 mm cracks started to appear more or less at the same load level as the previous specimens. The larger cracks, instead, formed at higher loads; 0.4 mm cracks, in particular, appeared just before the yielding load. Contrarily to what was seen in the previous beams, at the maximum load, a high number of cracks overcame the 0.4 mm threshold. Beam P3-A was characterized by an earlier appearance of both small and large cracks. Upon reaching the yielding load, half of the cracks had a crack opening bigger than 0.3 mm and more than a third overcame the 0.4 mm threshold. It is also possible to see that at the maximum load nearly every crack was wider than 0.3 mm. The lower performance of such a solution could be the result of some early damage in the strengthening layer, possibly caused by the weight of the concrete in its fresh state.

Beams FR-U1-M and FR-U1 had a slightly lower number of small cracks at the yielding and maximum load levels than P1. The 0.4 mm cracks, however, tended to dominate the crack patterns; they appeared at significantly lower loads and increased nearly linearly with the load. This could be the result of the higher tensile strength of the material, which successfully prevented the formation of small cracks while concentrating the elongation of the strengthening element in fewer points leading to a crack localization. With reference to beam FR-U2-A, the number of smaller cracks increased rapidly, shortly after their appearance, and slowed down in the later stages. The largest cracks had a fast appearing rate close to the reaching of the yielding load. Overall, this beam showed the lowest total number of cracks, most of them, however, had already overcome the 0.4 mm threshold at the yielding stage. Beam U1 exhibited a behaviour similar to beam P1. The smaller cracks appeared at relatively small loads with their number increasing nearly linearly, while the larger ones started to increase in number shortly before reaching the yielding load. The total number of cracks, however, is higher than specimen P1, both at the yielding and maximum load. Furthermore, the crack pattern is characterised by the presence of larger cracks, again both at the yielding and maximum load levels.

Beams FR-P2-B, FR-U2-B and P3-C were characterised by the highest number of cracks of the whole test series. In the case of specimen FR-P2-B, before reaching the yielding load, the crack pattern was characterised by the presence of many small cracks (0.1 mm and 0.2 mm) and only a few larger cracks. After reaching such load level the larger cracks increased rapidly, leading to a crack pattern characterised by the presence of mainly 0.4 mm cracks. Referring to beams FR-U2-B and P3-C, an earlier appearance of the larger cracks can be observed. Furthermore, at the maximum load, while 0.4 mm cracks are still predominant, a non-negligible amount of smaller cracks is present.

## 4. Discussion

**Figure 8:**
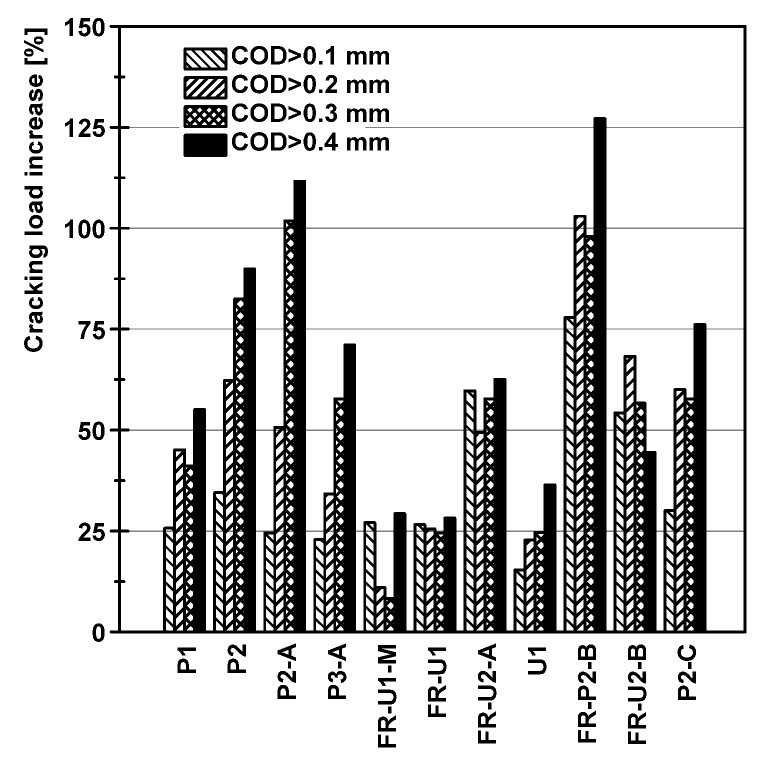
Increase in cracking load.

From the previously described results, several interesting aspects on the ability of TRC and F/TRC materials to control the crack formation and evolution on beams subjected to bending loads arise. The first considerations can be made on the variation of the load values at the reference crack openings. The results are reported in Table 3 and Figure 8 in terms of both load and load increase, calculated as the difference between the load of the strengthened beam ($P_{cr}$) and the reference beam ($P_{cr}^{REF}$) and divided by the cracking load of the reference beam (Equation (1)). The cracking load was defined as the load at which a crack has an opening overcoming one of the previously mentioned thresholds.

(1) $$\Delta P_{cr}=\frac{P_{cr}-P_{cr}^{REF}}{P_{cr}^{REF}}$$

All the tested strengthening solutions provided an increase in the cracking load of each crack typology. Higher increases in the cracking load are, in most cases, associated with the larger crack thresholds (0.3 mm and 0.4 mm). Focusing on the beams strengthened with HPC, it is possible to see how increasing the number of layers from one to two resulted in a significant increase in the cracking load, especially for the larger cracks. The installation of the threaded studs resulted in a cracking load increase for 0.3 mm and 0.4 mm cracks, but was less effective for the smaller cracks (0.1 mm and 0.2 mm). The addition of a third layer led to a lower increase in the cracking load. Once again, this phenomenon is attributed to the weight of the strengthening element that, while the concrete was still in its fresh state, might have experienced some damage.

The cracking load increase in the strengthening solutions where UHPC or UHPFRC was used was less effective. Comparing specimens FR-U1-M and FR-U1, it is possible to see that both of them achieved similar increases for 0.1 mm and 0.4 mm cracks. The increase for 0.2 mm and 0.3 mm cracks for beam FR-U1-M, instead was significantly lower. This phenomenon can be attributed to the lower viscosity that fresh UHPFRC exhibited when pumped, which, in turn, enabled better bonding performances. Specimen U1, on the other hand, showed a worse performance concerning 0.1 mm cracks but a higher load increase for 0.4 mm cracks, when compared to specimen FR-U1. The addition of a second textile layer, as in specimen FR-U2-A, resulted in a higher cracking load increase for every threshold.

Beam FR-P2-B showed the best results in terms of increase in cracking load, with an increase of more than 125% for 0.4 mm cracks. The results of specimen FR-U2-B are similar to specimen FR-U2-A, with a better performance in the case of 0.2 mm cracks and a worse one for 0.4 mm cracks. The cracking load increase in beam P2-C is similar to P2 and P2-A in terms of smaller cracks. The introduction of the bonded anchors, both in configuration FR-U2-B and P2-C, resulted in lower performance in the case of 0.3 mm and 0.4 mm cracks when compared to specimens FR-U2-A and P2-A, respectively. Analysing the load increase associated with 0.1 mm cracks a particular behaviour arises. Nearly all specimens exhibited very similar values of cracking load increase. The main outliers are the specimens strengthened with a combination of F/TRC and mechanical anchorages (beams FR-U2-A, FR-P2-B and FR-U2-B). This phenomenon suggests that such combination enables an earlier activation of the mechanical anchorage, making such solutions particularly suited for crack control in rather aggressive environments.

To further characterise the behaviour of the specimens, the crack patterns were analysed at two different discrete load levels. Such loads, defined approximately as one-half (150 kN) and two-thirds (200 kN) of the ultimate load of the reference beams, are meant to represent Serviceability Limit State (SLS) loading conditions. The 150 kN load should be representative of the normal usage of the structure, while the 200 kN load should represent a more demanding condition (e.g., increase in traffic loads which were not foreseeable during the design phase) which would justify a strengthening intervention.

### 4.1. Number of Cracks at the Reference Loads

**Figure 9:**
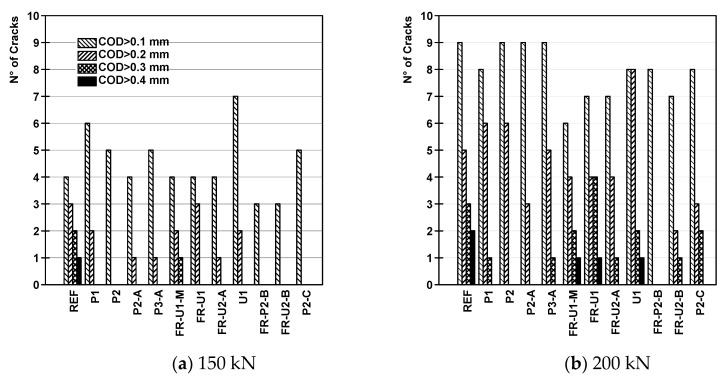
N° of cracks at reference loads.

Referring to Figure 9, it is possible to see that the strengthened beams exhibited, on average, a slightly higher number of 0.1 mm cracks at 150 kN than the reference beam. Cracks with a higher threshold appeared in a significantly lower number and, in the case of 0.4 mm cracks, did not appear at all. The total number of cracks for beams P1 to P3-A is in the same range as the reference beam, however, no cracks larger than 0.3 mm could be observed.

Beams FR-U1-M to FR-U2-A had the same number of 0.1 mm cracks of the reference beam at 150 kN. A lower number of 0.2 mm cracks was recorded for beams FR-U1-M and FR-U2-A. No 0.3 mm or 0.4 mm cracks could be observed, with the exception of specimen FR-U1-M, which had a single crack overcoming such threshold. Beam U1 had a significantly higher number of 0.1 mm cracks. Once again, no 0.3 mm crack or larger was recorded. The beams where the bonded anchors were installed exhibited only 0.1 mm cracks at 150 kN. Where short dispersed steel fibres were added to the cementitious matrix (FR-P2-B and FR-U2-B), a maximum of three cracks was recorded. From such results, it is clear that the use of TRC or F/TRC as strengthening solutions is able to increase the performance of the retrofitted elements at the SLS level, independently of the implemented solution. The use of bonded anchors, together with short dispersed fibres and a two-layer configuration, seems the most suited to protect structures against degradation.

At 200 kN, every beam recorded a significant increase in the number of cracks and, contrarily to what was observed at 150 kN, every strengthened specimen exhibited an equal or lower number of 0.1 mm cracks than the reference beam. Focusing on the reference beam, the increase was observed in every crack typology, with a less pronounced effect the bigger the crack. The specimens strengthened with HPC and without bonded anchors (from beam P1 to beam P3-A) had approximately the same number of 0.1 mm and 0.2 mm cracks as the reference beam; however, (P1 and P3-A) 0.3 mm cracks appeared only in two cases. Specimens FR-U1-M, FR-U1 and FR-U2-A showed a less pronounced crack increase for the smallest cracks, recording a total number of cracks lower than the reference beam. Once more, these specimens were characterised by the presence of larger cracks. Beam U1 showed a negligible increase in the number of 0.1 mm cracks with a width of 0.2 mm however significantly increased in numbers from 2 at 150 kN to 8 at 200 kN. Furthermore, every recorded crack showed an opening of at least 0.2 mm. The beams where the bonded anchors were installed also showed an important increase in the number of 0.1 mm cracks. The formation of the larger cracks was strongly reduced, in particular in the case of specimen FR-P2-B, where no crack overcame the 0.2 mm threshold. This phenomenon is probably due to the capability of the bonded anchors to effectively redistribute the stresses from the strengthening layers to the internal reinforcement of the beams.

These results confirm the effectiveness of TRC and F/TRC strengthening solutions, not only for ULS scenarios, but also at SLS levels where crack control can be a governing factor. Within this framework, it is thus possible to attribute to the beams strengthened with fine-grain HPC better results. The installation of the bonded anchors also proved to be an effective way of further increasing the performance. This is particularly evident in the case of beam FR-P2-B, where, at 200 kN, the specimen could still comply with the requirements of most international codes with crack opening smaller than 0.2 mm.

### 4.2. Maximum and Average Crack Opening at the Reference Loads

**Figure 10:**
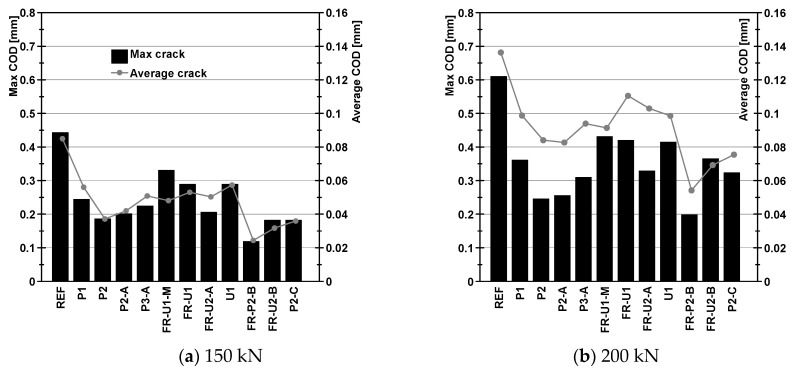
Maximum and average crack opening at reference loads.

Lastly, the results were analysed at the reference loads in terms of maximum and average crack opening (Figure 10). All retrofitted beams exhibited a lower maximum crack opening than the reference beam, especially at 200 kN. A reduction in such parameter can be seen between beams P1 and P2 and beams FR-U1 and FR-U2-A, showing that the addition of the second textile layer helps controlling crack opening. Once more, the presence of a third layer resulted in a larger maximum crack opening. The beams strengthened using UHPFRC or UHPC, on average, showed higher values of maximum crack opening both at 150 kN and 200 kN. The installation of the bonded anchors proved to be strongly effective in reducing the maximum crack opening at low load levels (150 kN). The strengthening system used in beam FR-P2-B, in particular, proved to be very effective, recording the smallest maximum crack opening of the whole series, both at 150 kN and 200 kN. The other two specimens where the bonded anchors were placed had a relatively low maximum crack opening at 150 kN, but were subjected to a strong increase when a load of 200 kN was reached, with a more evident effect in the case of beam FR-U2-B. Nevertheless, the maximum crack opening was nearly half of what was observed in the reference beam, showing that such solutions are not only viable options, but can also provide an important performance increase.

In terms of average crack opening, it can be seen that all the strengthened beams showed reduced values in comparison with the reference specimen. The best results were achieved when the bonded anchors were installed (FR-P2-B, FR-U2-B and P2-C) with the lowest values recorded by specimen FR-P2-B. Comparing them to specimens P1 to P3-A, it can be seen that the performance increase is particularly noticeable at 200 kN, probably due to a more effective stress redistribution. Specimens FR-U1-M to U1, while still showing a reduction in the average crack opening, resulted to be less effective. This can be attributed to the presence of a reduced number of cracks, which, however, resulted in a higher average crack opening.

These results confirm once again the suitability of TRC and F/TRC retrofitting strategies for the load increase and the control of the cracking behaviour in RC beams subjected to bending loads, thus enhancing their performance against environmental actions. Furthermore, it can be observed that fine-grain HPC exhibited generally better performances in reducing the crack width. The introduction of short threaded studs, as in configuration A, seemed to have a counter-productive effect in reducing the maximum crack opening. The longer bonded anchors, instead, proved to be more effective, especially in reducing the average crack opening at higher loads.

## 5. Conclusions

An experimental campaign, aimed at identifying the effect of TRC and F/TRC strengthening elements upon the cracking behaviour of beams subjected to bending loads, was performed at the structural lab of Carinthia University of Applied Sciences. Different strengthening solutions, varying in terms of the number of textile layers, cementitious materials and configuration of mechanical anchorages, were analysed. From these results, the following conclusions can be made:

- Strengthening reinforced concrete beams with TRC or F/TRC materials proved to be an effective and viable solution, not only for retrofitting, but also for a reduction in damage and crack opening control, thus increasing their performance.
- Each strengthening solution resulted in an increase in the cracking load (between 8.3% and 127.1%), depending on the number of textile layers, cementitious matrix, and type and arrangement of mechanical anchorages.
- The combination of short dispersed steel fibres and mechanical anchorage devices has the highest potential in increasing the cracking load (between 59.7% and 77.9%) associated with very small cracks (0.1 mm).
- The use of fine-grain HPC in combination with textile reinforcement proved to outperform UHPFRC in terms of cracking load increase, number of large cracks and ability to reduce both the maximum and average crack opening.
- Bonded anchors, used as end anchorage of the strengthening TRC and F/TRC layer, were very effective in preventing the formation of larger cracks and in reducing both the maximum and average crack opening. They also provided the best performance increase in terms of cracking load and the number of cracks at low load levels only when they were combined with short dispersed steel fibres. Furthermore, all beams strengthened with bonded anchors were able to achieve the highest gain in maximum strength compared to beams without those anchoring elements.

## Figures and Tables

**Table 1:** Strengthening solutions and characteristics of the cementitious materials.

Specimen ID	Casting Method *	N° of Layers/Anchorage	Cementitious Matrix	Compressive Strength [MPa]	Tensile Strength [MPa]
REF1	–	–	–	55.2	–
P1	L	1/–	HPC	88.7	4.8
P2	L	2/–	HPC	81.9	4.8
P2-A	L	2/A	HPC	80.8	5.9
P3-A	L	3/A	HPC	92.7	6.6
FR-U1-M	M	1/–	UHPFRC	169	13.0
FR-U1	P	1/–	UHPFRC	159.2	11.8
FR-U2-A	P	2/A	UHPFRC	146.4	13.3
U1	P	1/–	UHPC	184.4	5.2
FR-P2-B	L	2/B	HPFRC	89.7	9.3
FR-U2-B	P	2/B	UHPFRC	184.1	14.3
P2-C	L	2/C	HPC	88.8	3.3
REF2	–	–	–	59.1	–

* L: Lamination; M: Manual Pouring; P: Pumping.

**Table 2:** Yielding Loads, Failure Loads and Failure Modes of the tested specimens.

Specimen ID	Cracking Load (kN) ^1^	Yielding Load (kN)	Maximum Load (kN)	Peak Load Increase (%)	Failure Mode
REF-1	73.66	275.78	316.87		–
P1	92.62	297.79	337.68	6.29	d
P2	99.08	324.08	367.95	15.81	d/e
P2-A	91.76	321.90	387.71	22.03	d/e
P3-A	90.53	357.15	416.94	31.23	d/e
FR-U1-M	93.64	317.67	370.41	16.59	c
FR-U1	93.27	316.56	370.46	16.61	a
FR-U2-A	117.66	342.36	370.35	16.57	e
U1	84.97	304.87	362.13	13.98	a/d
FR-P2-B	131.01	342.86	447.10	40.73	b/d
FR-U2-B	113.65	356.74	470.24	48.01	b/c
P2-C	95.80	334.57	447.28	40.78	b/d
REF-2	–	274.53	318.54		–

^1^ The cracking load was defined as the load at which a 0.1 mm crack could be detected.

**Table 3:** Cracking loads and relative increase.

Beam ID	Load at Crack Opening (kN)				Load Increase (%)			
	0.1 mm	0.2 mm	0.3 mm	0.4 mm	0.1 mm	0.2 mm	0.3 mm	0.4 mm
REF-1	73.66	100.54	121.69	143.12	–	–	–	–
P1	92.62	145.92	171.80	222.00	25.7	45.1	41.2	55.1
P2	99.08	163.21	222.10	271.80	34.5	62.3	82.5	89.9
P2-A	91.76	151.49	245.6	302.87	24.6	50.7	101.8	111.6
P3-A	90.53	134.95	191.94	244.88	22.9	34.2	57.7	71.1
FR-U1-M	93.64	111.67	131.83	185.12	27.1	11.1	8.3	29.3
FR-U1	93.27	126.21	151.57	183.52	26.6	25.5	24.6	28.2
FR-U2-A	117.66	150.25	191.99	232.65	59.7	49.4	57.8	62.6
U1	84.97	123.40	151.68	195.27	15.4	22.7	24.6	36.4
FR-P2-B	131.01	204.07	240.89	325.05	77.9	103.0	98.0	127.1
FR-U2-B	113.65	169.20	190.67	206.83	54.3	68.3	56.7	44.5
P2-C	95.80	160.92	192.00	252.06	30.1	60.1	57.8	76.1

## Data Availability

All data that support the findings of this study are available from the corresponding author upon reasonable request.

## References

[1] American Society of Civil Engineers (2013). Report Card for America’s Infrastructure.

[2] Bell B., Network Rail (2004). European Railway Bridge Demography. http://bridgeforum.org/files/pub/2006/sustainable-bridges/WP1-02-T-040531-R-Deliverable%20D%201.2.pdf.

[3] Bundesministerium Für Verkehr und Digitale Infrastruktur (German Federal Ministry of Transport and Digital Infrastructure), Stand der Modernisierung von Straßenbrücken der Bundesfernstraßen 2018. https://www.bmvi.de/SharedDocs/DE/Anlage/StB/bericht-stand-der-modernisierung-von-strassenbruecken-2018.pdf?__blob=publicationFile.

[4] Awani O., El-Maaddawy T., Ismail N. (2017). Fabric-Reinforced Cementitious Matrix: A Promising Strengthening Technique for Concrete Structures. Constr. Build. Mater..

[5] Nobili A., Falope F.O. (2017). Impregnated Carbon Fabric-Reinforced Cementitious Matrix Composite for Rehabilitation of the Finale Emilia Hospital Roofs: Case Study. J. Compos. Constr..

[6] Bencardino F., Carloni C., Condello A., Focacci F., Napoli A., Realfonzo R. (2018). Flexural Behaviour of RC Members Strengthened with FRCM: State-of-the-Art and Predictive Formulas. Compos. Part B Eng..

[7] Koutas L.N., Tetta Z., Bournas D.A., Triantafillou T.C. (2019). Strengthening of Concrete Structures with Textile Reinforced Mortars: State-of-the-Art Review. J. Compos. Constr..

[8] Tarque N., Salsavilca J., Yacila J., Camata G. (2019). Multi-Criteria Analysis of Five Reinforcement Options for Peruvian Confined Masonry Walls. Earthq. Struct..

[9] Wakjira T.G., Ebead U. (2019). Experimental and Analytical Study on Strengthening of Reinforced Concrete T-Beams in Shear Using Steel Reinforced Grout (SRG). Compos. Part B Eng..

[10] Salsavilca J., Yacila J., Tarque N., Camata G. (2020). Experimental and Analytical Bond Behaviour of Masonry Strengthened with Steel Reinforced Grout (SRG). Constr. Build. Mater..

[11] Funari M.F., Verre S. (2021). The Effectiveness of the DIC as a Measurement System in SRG Shear Strengthened Reinforced Concrete Beams. Crystals.

[12] Li B., Xiong H., Jiang J., Dou X. (2019). Tensile Behavior of Basalt Textile Grid Reinforced Engineering Cementitious Composite. Compos. Part B Eng..

[13] Deng M., Dong Z., Zhang C. (2020). Experimental Investigation on Tensile Behavior of Carbon Textile Reinforced Mortar (TRM) Added with Short Polyvinyl Alcohol (PVA) Fibers. Constr. Build. Mater..

[14] Dong Z., Deng M., Zhang C., Zhang Y., Sun H. (2020). Tensile Behavior of Glass Textile Reinforced Mortar (TRM) Added with Short PVA Fibers. Constr. Build. Mater..

[15] Li T., Deng M., Dong Z., Zhang Y., Zhang C. (2020). Masonry Columns Confined with Glass Textile-Reinforced High Ductile Concrete (TRHDC) Jacket. Eng. Struct..

[16] Mészöly T., Ofner S., Randl N. (2020). Effect of Combining Fiber and Textile Reinforcement on the Flexural Behavior of UHPC Plates. Adv. Mater. Sci. Eng..

[17] Yang X., Gao W.-Y., Dai J.-G., Lu Z.-D. (2020). Shear Strengthening of RC Beams with FRP Grid-Reinforced ECC Matrix. Compos. Struct..

[18] Zheng Y.-Z., Wang W.-W., Mosalam K.M., Fang Q., Chen L., Zhu Z.-F. (2020). Experimental Investigation and Numerical Analysis of RC Beams Shear Strengthened with FRP/ECC Composite Layer. Compos. Struct..

[19] Li T., Deng M., Jin M., Dong Z., Zhang Y. (2021). Performance of Axially Loaded Masonry Columns Confined Using Textile Reinforced Concrete (TRC) Added with Short Fibers. Constr. Build. Mater..

[20] Rossi E., Randl N., Mészöly T., Harsányi P. (2021). Flexural Strengthening with Fiber-/Textile-Reinforced Concrete. ACI Struct. J..

[21] Rossi E., Randl N., Harsányi P., Mészöly T. (2021). Overlapped Joints in Textile Reinforced Concrete with UHPC Matrix: An Experimental Investigation. Mater. Struct..

[22] Yin S., Lü H., Xu S. (2013). Properties and Calculation of Normal Section Bearing Capacity of RC Flexural Beam with Skin Textile Reinforcement. J. Cent. South Univ..

[23] Verbruggen S., Aggelis D.G., Tysmans T., Wastiels J. (2014). Bending of Beams Externally Reinforced with TRC and CFRP Monitored by DIC and AE. Compos. Struct..

[24] Verbruggen S., Tysmans T., Wastiels J. (2014). TRC or CFRP Strengthening for Reinforced Concrete Beams: An Experimental Study of the Cracking Behaviour. Eng. Struct..

[25] Verbruggen S., Tysmans T., Wastiels J. (2016). Bending Crack Behaviour of Plain Concrete Beams Externally Reinforced with TRC. Mater. Struct..

[26] Park J., Park S.-K., Hong S. (2020). Experimental Study of Flexural Behavior of Reinforced Concrete Beam Strengthened with Prestressed Textile-Reinforced Mortar. Materials.

[27] Mészöly T., Randl N. Derivation of constitutive law for UHPFRC using DIC system. Proceedings of the AFGC-ACI-fib-RILEM International Symposium on Ultra-High Performance Fibre-Reinforced Concrete.

[28] Mészöly T., Randl N. (2018). An advanced approach to derive the constitutive law of UHPFRC. Archit. Civ. Eng. Environ..

[29] Mészöly T., Randl N. (2018). Shear Behavior of Fiber-Reinforced Ultra-High Performance Concrete Beams. Eng. Struct..

[30] Randl N., Harsányi P. (2018). Developing Optimized Strengthening Systems for Shear-Deficient Concrete Members. Struct. Concr..

[31] (2018). Solidian GRID Q142/142-CCE-25 Technical Data Sheet, Version: 180703.

[32] Randl N., Steiner T., Ofner S., Baumgartner E., Mészöly T. (2014). Development of UHPC Mixtures from an Ecological Point of View. Constr. Build. Mater..

[33] Ricker M., Häusler F., Randl N. (2017). Punching Strength of Flat Plates Reinforced with UHPC and Double-Headed Studs. Eng. Struct..

[34] Deutsches Institut für Bautechnik Verfahren zur Verstärkung von Stahlbeton Mmit TUDALIT (Textilbewehrter Beton) 2016, Technical Approval Number: Z-31.10-182. https://www.irbnet.de/daten/bzp/2FF929E209/bzp-bfi_3146144.pdf.

[35] Graybeal B.A. (2006). Material Property Characterization of Ultra-High Performance Concrete.

[36] Wang L., He T., Zhou Y., Tang S., Tan J., Liu Z., Su J. (2021). The Influence of Fiber Type and Length on the Cracking Resistance, Durability and Pore Structure of Face Slab Concrete. Constr. Build. Mater..

[37] Wang L., Jin M., Guo F., Wang Y., Tang S. (2021). Pore structural and fractal analysis of the influence of fly ash and silica fume on the mechanical property and abrasion resistance of concrete. Fractals.

[38] European Committee for Standardization (2004). EN 1992-1-1, Eurocode 2: Design of Concrete Structures—Part 1-1: General Rules and Rules for Buildings.

[39] François R., Arliguie G. (1998). Influence of Service Cracking on Reinforcement Steel Corrosion. J. Mater. Civ. Eng.

[40] Castel A., Vidal T., François R., Arliguie G. (2003). Influence of Steel-Concrete Interface Quality on Reinforcement Corrosion Induced by Chlorides. Mag. Concr. Res.

[41] Michel A., Solgaard A.O.S., Pease B.J., Geiker M.R., Stang H., Olesen J.F. (2013). Experimental Investigation of the Relation between Damage at the Concrete-Steel Interface and Initiation of Reinforcement Corrosion in Plain and Fibre Reinforced Concrete. Corros. Sci..

[42] ACI Committee 224 (2002). ACI 224R-01 Conctrol of Cracking in Concrete Structures.

[43] CEB-FIP (2013). Fib Model Code for Concrete Structures 2010.

